# Deficiency in the anti‐aging gene Klotho promotes aortic valve fibrosis through AMPKα‐mediated activation of RUNX2

**DOI:** 10.1111/acel.12494

**Published:** 2016-05-31

**Authors:** Jianglei Chen, Yi Lin, Zhongjie Sun

**Affiliations:** ^1^ Department of PhysiologyCollege of MedicineUniversity of Oklahoma Health Sciences CenterOklahoma CityOK73104USA

**Keywords:** AMPKα, aortic valve, aortic valve interstitial cells, fibrosis, Klotho, RUNX2

## Abstract

Fibrotic aortic valve disease (FAVD) is an important cause of aortic stenosis, yet currently there is no effective treatment for FAVD due to its unknown etiology. The purpose of this study was to investigate whether deficiency in the anti‐aging Klotho gene (*KL*) promotes high‐fat‐diet‐induced FAVD and to explore the underlying molecular mechanism. Heterozygous Klotho‐deficient (*KL* ^+/−^) mice and WT littermates were fed with a high‐fat diet (HFD) or normal diet for 13 weeks, followed by treatment with the AMPKα activator (AICAR) for an additional 2 weeks. A HFD caused a greater increase in collagen levels in the aortic valves of *KL* ^*+/−*^ mice than of WT mice, indicating that Klotho deficiency promotes HFD‐induced aortic valve fibrosis (AVF). AMPKα activity (pAMPKα) was decreased, while protein expression of collagen I and RUNX2 was increased in the aortic valves of *KL* ^+/−^ mice fed with a HFD. Treatment with AICAR markedly attenuated HFD‐induced AVF in *KL* ^+/−^ mice. AICAR not only abolished the downregulation of pAMPKα but also eliminated the upregulation of collagen I and RUNX2 in the aortic valves of *KL* ^+/−^ mice fed with HFD. In cultured porcine aortic valve interstitial cells, Klotho‐deficient serum plus cholesterol increased RUNX2 and collagen I protein expression, which were attenuated by activation of AMPKα by AICAR. Interestingly, silencing of RUNX2 abolished the stimulatory effect of Klotho deficiency on cholesterol‐induced upregulation of matrix proteins, including collagen I and osteocalcin. In conclusion, Klotho gene deficiency promotes HFD‐induced fibrosis in aortic valves, likely through the AMPKα–RUNX2 pathway.

## Introduction

In 2010, more than 15 000 deaths were directly caused by aortic valve disease (AVD) in the USA, making it the second‐leading cause of cardiovascular mortality (Go *et al*., 2014). The prevalence of moderate or severe aortic stenosis in the general population > 75 years old is 2.8%. While approximately 50% of patients with severe aortic stenosis are referred for aortic valve replacement (AVR), only 40% are actually admitted for AVR. The development of transcatheter AVR provides a less‐invasive approach than surgical replacement. However, this option is currently only available for patients who were not surgical candidates for AVR, and the 2‐year mortality and hospitalization rates are > 50% (Go *et al*., 2014). The prevalence of AVD is an increasing burden on the healthcare system as global life expectancy increases (Nkomo *et al*., 2006; Go *et al*., 2014).

For decades, AVD was thought to be a passive process involving fatigue or deterioration of the valve with age. Currently, AVD is viewed as an active, cellular‐driven disease that is not an inevitable consequence of aging (Rajamannan *et al*., 2011). However, no drug therapies have been developed specifically for AVD, and although AVD shares several risk factors and mechanisms with vascular diseases (e.g., atherosclerosis), there are fundamental differences between arteries and the aortic valve with respect to disease mechanisms and response to therapeutic interventions (Weiss *et al*., 2013). Aortic valve fibrosis (AVF) is an important pathological process that eventually leads to aortic valve stiffening and aortic stenosis. Unfortunately, the pathological mechanisms driving AVF are poorly understood.

Klotho (*KL*) was originally identified as a putative aging‐suppressor gene and is predominately expressed in kidneys and the brain choroid plexus (Kuro‐o *et al*., 1997). It extends lifespan and accelerates aging when disrupted in mice (Kuro‐o *et al*., 1997; Xu & Sun, 2015). Specifically, Klotho‐deficient mice display multiple pathologies resembling human aging, such as endothelial dysfunction, soft tissue calcification, progressive atherosclerosis, and shortened lifespan (Kuro‐o, 2009, 2011). Klotho protein is found in the blood (Xu & Sun, 2015), and its serum level declines with the normal aging process (Xiao *et al*., 2004; Xu & Sun, 2015). By age 80, the serum level of Klotho is about a half of what it was at age 40 (Xiao *et al*., 2004). By contrast, the prevalence of AVD and aortic stenosis increases with age (Lindroos *et al*., 1993). However, whether a reduction in Klotho contributes to AVF has never been investigated. A reduction in the level of Klotho is also observed in chronic kidney disease, hypertension, and diabetes mellitus (Wang *et al*., 2012; Chen *et al*., 2015; Lin & Sun, 2015a,c).

A hallmark of AVD initiation is fibrotic collagen accumulation and calcific nodule formation within the leaflets, which exacerbate the loss of tissue compliance and function, ultimately leading to aortic stenosis (Weiss *et al*. 2013). However, whether this fibrotic response is inseparable from the formation of calcific nodules or whether valve fibrosis and calcification are parallel processes during the development of AVD remains uncertain. On the other hand, fibrotic collagen accumulation, which leads to thickened and stiffened aortic valve leaflets and subsequent degeneration of valve function, could cause aortic stenosis (Miller *et al*., 2011). The epidemiological risk factors of AVD resemble those of atherosclerosis, including elevated serum cholesterol, hypertension, smoking, diabetes, and male gender (Lindroos *et al*., 1994; Stewart *et al*., 1997). Low‐density lipoprotein accumulation was found in stenotic aortic valves in humans, and dietary hypercholesterolemia induced aortic valve stenosis in small animal models (Weiss *et al*. 2013). However, several clinical trials targeting cholesterol using lipid‐lowering therapy did not slow obvious effects on the progression of AVD (Cowell *et al*., 2005; Houslay *et al*., 2006; Rossebo *et al*., 2008; Chan *et al*., 2010), and the beneficial effect of cholesterol‐lowering treatment is limited (Rosenhek *et al*., 2004). Although the failure of these clinical studies may be multifactorial, it suggests that key pathological factors that promote AVD remain to be determined. Nevertheless, it is currently believed that high cholesterol levels are an early factor that contributes to the development of AVD (Choi *et al*., 2015). In this study, we investigated whether Klotho gene deficiency promotes fibrotic AVD (FAVD) in mice fed with a high‐fat diet (HFD).

Runt‐related transcription factor 2 (RUNX2, also known as a core‐binding factor subunit alpha‐1, CBFα1) is encoded by the *RUNX2* gene. RUNX2 has been identified as a ‘master gene’ in the differentiation of osteoblasts, serving as a key transcription factor that regulates extracellular matrix (ECM) gene products (e.g., osteocalcin, OCN) (Lee *et al*., 2000; Tu *et al*., 2008). OCN is secreted by osteoblasts, it is believed to play a role in the body's metabolic regulation, and it is pro‐osteoblastic (bone building; Lee *et al*., 2007). OCN is therefore often used as a marker of bone formation.

AMP‐dependent protein kinase (AMPK) is a serine/threonine protein kinase that serves as an energy sensor in the regulation of cellular metabolism. Recent studies showed that AMPK is expressed in vascular endothelial cells and that its activation improves endothelial function by suppressing oxidative stress (Zou & Wu, 2008; Wang *et al*., 2010). The major isoform of AMPK in endothelial cells is AMPKα1β1γ1, with α1 being the catalytic subunit. Downregulation of AMPKα leads to vascular dysfunction. Fortunately, an analog of AMP, 5‐amino‐1‐β‐D‐ribofuranosyl‐imidazole‐4‐carboxamide (AICAR, also known as ZMP), stimulates AMPK activity. AICAR does not perturb the cellular content of ATP, ADP, or AMP but activates AMPKα due to increased phosphorylation (Thr‐172; Corton *et al*., 1995). In this study, we assessed whether activation of AMPKα by AICAR attenuates the AVF‐promoting effect of Klotho deficiency in mice fed with HFD.

## Methods

### Animal studies

Heterozygous *KL* ^*+/−*^ mutant mice with the 129/Sv background were kindly provided by Dr. Kuro‐o (Kuro‐o *et al*., 1997). This study was approved by the Institutional Animal Care and Use Committee at the University of Oklahoma Health Sciences Center. Immunohistochemical (IHC) procedures were performed as described in our previous studies (Crosswhite *et al*., 2014; Chen *et al*., 2015; Lin & Sun, 2015a,c; Zhou *et al*., 2015b). Western blotting was performed as described in our previous studies (Goetz *et al*., 2010; Belting *et al*., 2012; Chen *et al*., 2015; Lin & Sun, 2015b,c; Zhou *et al*., 2015a; Lin *et al*., 2016). For details, see the Appendix S1 (Supporting information).

### Statistical analysis

Data were analyzed using one‐way analysis of variance (ANOVA). The Newman–Keuls procedure was used to assess differences between means. Data were expressed as mean ± SEM. *P* < 0.05 was considered significant.

## Results

### Klotho deficiency downregulated AMPKα activity and promoted fibrotic formation in aortic valves in mice fed with a HFD

**Figure 1:**
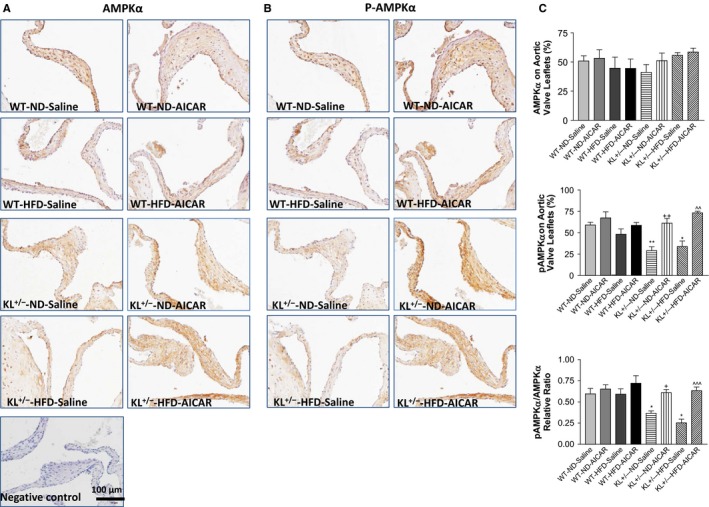
Klotho efficiency downregulated AMPKα activity in aortic valves in mice fed with a high‐fat diet (HFD). Immunohistochemical (IHC) staining of AMPKα (A) and pAMPKα (B) in the aortic valves of wild‐type and Klotho‐deficient (*KL* ^*+/−*^) mice after a 13‐week HFD followed by treatment with AICAR for 2 weeks. AMPKα and pAMPKα were stained brown. (C) Quantification of AMPKα and pAMPKα levels and their ratio (*N* = 4–6). Data = means ± SEM. *P <* *0.05, **P <* *0.01 vs. WT‐ND‐Saline; ^+^ *P* < 0.05, ^++^ *P* < 0.01 vs. *KL* ^+/−^‐ND‐Saline; ^*P* < 0.05, ^^*P* < 0.01, ^^^*P* < 0.001 vs. *KL* ^+/−^‐HFD‐Saline.

To evaluate whether Klotho deficiency plays a role in the development of AVF, we fed *KL* ^*+/−*^ mice with a HFD for 13 weeks, followed by treatment with AICAR for an additional 2 weeks. A HFD increased total blood cholesterol levels in both WT and *KL* ^+/−^ mice to the same extent (data not shown). Immunohistochemical staining showed that Klotho deficiency and/or a HFD did not change the basal AMPKα expression level in aortic valves (Fig. 1A,C, upper panel). Interestingly, Klotho deficiency plus HFD significantly decreased phosphorylation of AMPKα (pAMPKα, Thr172) in the aortic valves (Fig. 1B,C, middle panel), suggesting that Klotho deficiency downregulates AMPKα activity. The decreased ratio of pAMPKα/AMPKα also suggested a decrease in AMPKα activity (Fig. 1C, lower panel). Treatment with AICAR rescued the downregulation of AMPKα activity in *KL* ^*+/−*^ mice fed with a HFD (Fig. 1B,C).

**Figure 2:**
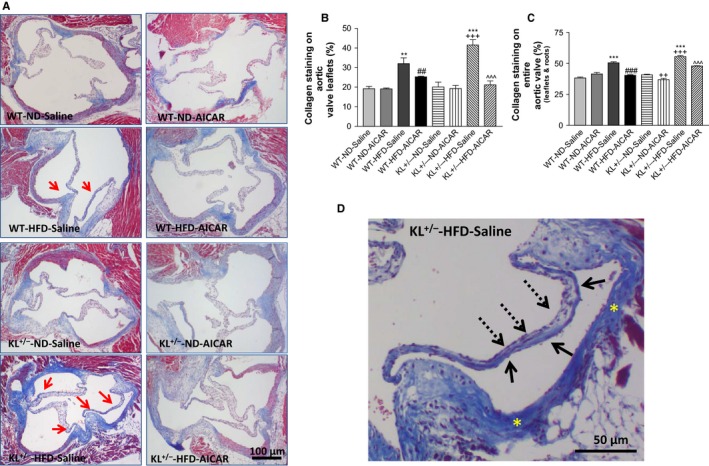
Klotho deficiency promoted fibrotic formation in aortic valves *via* downregulation of AMPKα activity in mice fed with a HFD. (A) Masson's trichrome staining of aortic valves of wild‐type and Klotho‐deficient (*KL* ^*+/−*^) mice after a 15‐week HFD feeding. Collagen deposition (blue) is markedly increased in the leaflets of *KL* ^*+/−*^ mice fed with a HFD. The red arrows indicate collagen deposition on the surface of the leaflets. (B) Quantification of collagen level in the leaflets (*N* = 4–6). (C) Quantification of collagen levels of the entire aortic valve region including the aortic root (*N* = 4). Data = means ± SEM. **P < 0.01, ***P < 0.001 vs. WT‐ND‐Saline; ^##^ *P* < 0.01, ^###^ *P *<* *0.001 vs. WT‐HFD‐Saline; ^++^ *P* < 0.001, ^+++^ *P* < 0.0001 vs. *KL* ^*+/−*^‐ND‐Saline; ^^^*P* < 0.001 vs. *KL* ^*+−/*^‐HFD‐Saline. (D) Higher magnification of aortic valves in a *KL* ^*+/−*^ mouse fed with HFD‐Saline, which shows asymmetrical sclerosis of aortic valves. The collagen deposition preferentially accumulated on the aortic surface (solid arrows) compared with the ventricular surface of the leaflets (dashed arrows). The yellow asterisks indicate severe mural fibrosis in aortic valves.

Masson trichrome staining showed a marked increase in collagen deposition on the aortic valves of *KL* ^*+/−*^ mice fed with a HFD (Fig. 2A–C). A significant increase in collagen was found on the leaflets (Fig. 2A,B) and root regions of aortic valves (Fig. 2A,C). AICAR treatment significantly reduced collagen deposition on the aortic valves (Fig. 2B,C). The aortic valves of *KL* ^*+/−*^ mice fed with a HFD showed typical pathological changes of valve sclerosis and stenosis, such as mural fibrosis (Fig. 2D, yellow asterisk), AVF (red arrows, Fig. 2A), and asymmetrical sclerosis of the leaflets (Fig. 2D, black arrows). Collagen preferentially accumulated on the aortic surface of the valve leaflets (solid arrows) compared with the ventricular surface (dashed arrows).

**Figure 3:**
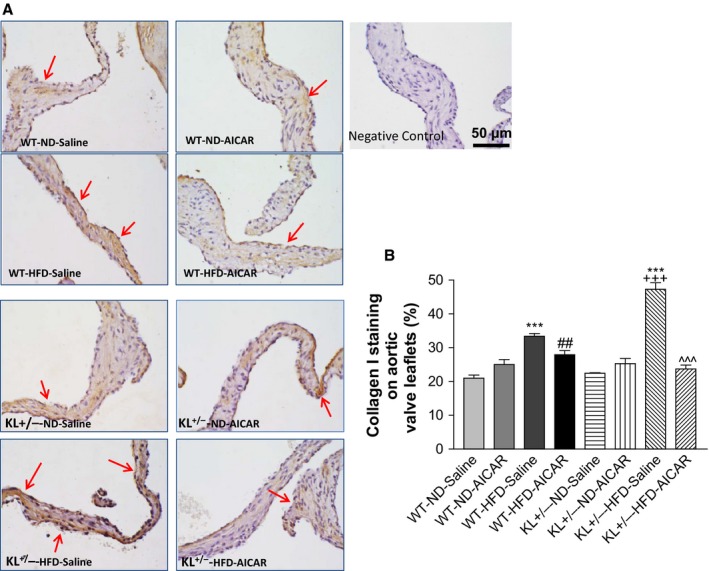
Klotho deficiency promoted upregulation of collagen I expression in aortic valves *via* downregulation of AMPKα in mice fed with HFD. (A) IHC staining of type I collagen (also known as collagen I) in the aortic valves of WT and Klotho‐deficient (*KL* ^*+/−*^) mice fed with a HFD for 13 weeks followed by treatment with AICAR for an additional 2 weeks. The collagen I deposition (brown) is significantly increased in the leaflets in *KL* ^*+/−*^ mice fed with a HFD. Red arrows indicate collagen I staining (brown color) on the surface of the leaflets. (B) Quantification of collagen I level in leaflets (*N* = 4–6). Data = means ± SEM. ***P < 0.001 vs. WT‐ND‐Saline; ^##^ *P* < 0.01 vs. WT‐HFD‐Saline; ^+++^ *P* < 0.001 vs. *KL* ^*+/−*^‐ND‐Saline, ^^^*P* < 0.001 vs. *KL* ^*+/−*^‐HFD‐Saline.

Immunohistochemical staining further demonstrated that type I collagen (also known as collagen I) expression was upregulated in the aortic valve in mice fed with a HFD, especially in *KL* ^*+/−*^ mice (Fig. 3A,B). This result suggests that AVF was mainly due to upregulation of collagen I. AICAR treatment abolished type I collagen accumulation in the aortic valve in *KL* ^*+/−*^ mice (Fig. 3A,B), suggesting that downregulation of AMPKα activity mediates Klotho deficiency‐induced upregulation of collagen I in the aortic valve.

### Klotho deficiency increased RUNX2 expression in the aortic valve *via* downregulation of AMPKα in mice fed with HFD

**Figure 4:**
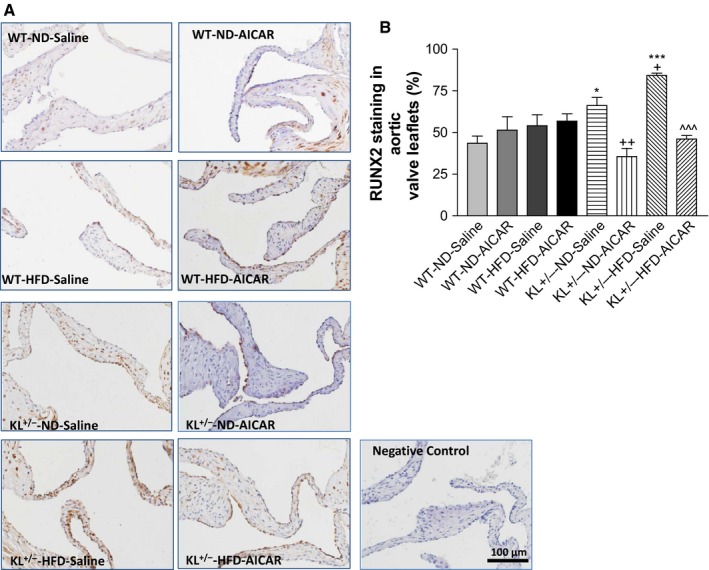
Klotho deficiency increased RUNX2 expression in aortic valves *via* downregulation of AMPKα in mice fed with a HFD. (A) IHC staining of RUNX2 on the aortic valves of wild‐type and Klotho‐deficient (*KL* ^*+/−*^) mice fed with a HFD for 13 weeks followed by treatment with AICAR for an additional 2 weeks. The RUNX2 staining (brown) was significantly increased in the leaflets of *KL* ^*+/−*^ mice fed with a HFD. (B) Quantification of RUNX2 expression in the leaflets (*N* = 4–6). Data = means ± SEM. *P < 0.05, ***P < 0.001 vs. WT‐ND‐Saline; ^++^ *P* < 0.01 vs. *KL* ^*+/−*^‐ND‐Saline, ^^^*P* < 0.001 vs. *KL* ^*+/–*^‐HFD‐Saline.

RUNX2 is a member of the RUNX family of transcription factors, which are involved in osteoblast differentiation and skeletal morphogenesis. IHC staining of RUNX2 in the aortic valve showed that RUNX2 was expressed in the interstitial cells in the aortic valve region (Fig. 4A). RUNX2 protein levels were significantly increased in *KL* ^*+/−*^ mice and especially in those fed with a HFD (Fig. 4A,B). Treatment with AICAR abolished the downregulation of RUNX2 expression in *KL* ^*+/−*^ mice fed with a HFD, suggesting that Klotho deficiency‐induced upregulation of RUNX2 is mediated by downregulation of AMPKα. Unexpectedly, Alizarin red staining showed that there was no obvious calcification in aortic valves (Fig. S1).

### Klotho deficiency upregulated RUNX2 and collagen I protein expression in PAVICs

**Figure 5:**
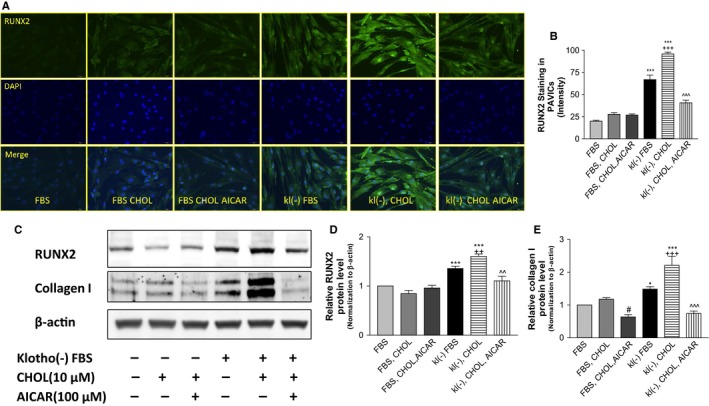
Upregulation of RUNX2 PAVICs treated with Klotho‐deficient FBS and/or cholesterol. Confluent cells were treated with Klotho‐deficient FBS and/or cholesterol for 24 h. (A) IHC for antibodies against RUNX2 (blue, DAPI; green, Runx2). (B) Semi‐quantification of RUNX2 in PAVICs (*N* = 10). (C) Western blot analysis of RUNX2 and collagen I. (D) Semi‐quantification of RUNX2 in Western blot. (E) Semi‐quantification of collagen I. Data were first normalized with β‐actin and then calculated as fold change of the FBS group. Data = means ± SEM. *P < 0.05, ***P < 0.001 vs. cells treated with normal FBS; ^#^ *P* < 0.05 vs. cells treated with normal FBS plus 10 μm cholesterol (FBS, CHOL); ^++^ *P* < 0.01, ^+++^ *P* < 0.001 vs. cells treated with Klotho‐deficient FBS (kl(–) FBS); ^^*P* < 0.01, ^^^*P* < 0.001 vs. cells treated with Klotho‐deficient FBS plus 10 μm cholesterol (kl(–) CHOL). *N* = 3 independent experiments. FBS, fetal bovine serum. kl(–), Klotho‐deficient FBS.

Due to the limited tissue size of mouse aortic valves, we used primary porcine aortic valve interstitial cells (PAVICs) for further mechanistic studies. These cells were cultured in Klotho‐deficient FBS (~50% secreted Klotho was removed from normal FBS through immunoprecipitation with the Klotho antibody (Fan & Sun, 2016). Immunofluorescent staining showed a marked increase in RUNX2 expression in the Klotho‐deficient, FBS‐treated cells, indicating that Klotho deficiency upregulates RUNX2 protein levels in PAVICs (Fig. 5A,B). The addition of cholesterol to the medium further enhanced Klotho deficiency‐induced upregulation of RUNX2. Interestingly, treatment with AICAR nearly abolished upregulation of RUNX2 expression in PAVICs treated with Klotho‐deficient FBS and cholesterol (Fig. 5A,B).

Interestingly, Klotho deficiency upregulated RUNX2 protein expression, which was further exacerbated by cholesterol (Fig. 5C,D). By contrast, activation of AMPKα by AICAR almost abolished the upregulation of RUNX2. The level of type I collagen, a major ECM protein, was increased significantly in the medium when cells were treated with Klotho‐deficient FBS and was further enhanced by cholesterol in PAVICs (Fig. 5C,E). This result suggests that Klotho deficiency increased collagen synthesis, which was exacerbated by cholesterol. By contrast, AICAR abolished the upregulation of collagen I expression induced by Klotho deficiency and cholesterol (Fig. 5C,E).

### Knockdown of RUNX2 abolished the upregulation of collagen I and OCN protein expression in PAVICs treated with Klotho‐deficient FBS and cholesterol

**Figure 6:**
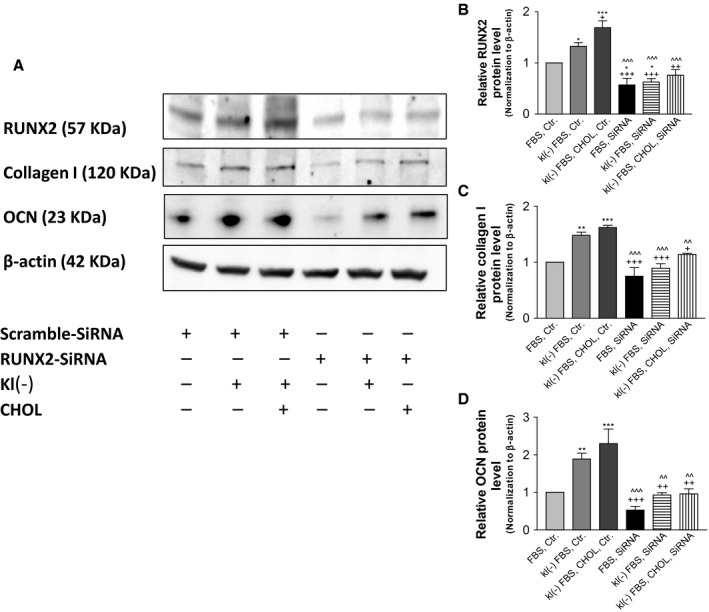
Knockdown of RUNX2 abolished upregulation of collagen I and OCN protein expression in PAVICs treated with Klotho‐deficient FBS and cholesterol. Confluent cells were first transfected with RUNX2 siRNA or scramble siRNA for 48 h and incubated with Klotho‐deficient FBS and cholesterol for 24 h. (A) Western blot analysis of collagen I, RUNX2, and OCN. (B–D) Quantification (*N* = 3) of Western blot of collagen I, RUNX2, and OCN in PAVICs. Data were first normalized with β‐actin and then calculated as fold change of the FBS, Ctr. group. Data = means ± SEM. *P < 0.05, **P < 0.01, ***P < 0.001 vs. normal FBS plus control siRNA (FBS Ctr); ^+^ *P* < 0.05, ^++^ *P* < 0.01, ^+++^ *P* < 0.001 vs. cells treated with Klotho‐deficient FBS plus control siRNA (kl(–) FBS Ctr); ^^*P* < 0.01, ^^^*P* < 0.001 vs. cells treated with Klotho‐deficient serum plus 10 μm cholesterol plus control siRNA (kl(–) FBS CHOL Ctr). *N* = 3 independent experiments. FBS, fetal bovine serum; kl(–), Klotho‐deficient FBS; Ctr, control siRNA; siRNA, RUNX2 siRNA.

To determine whether RUNX2 is required for the upregulation of collagen I induced by Klotho deficiency and cholesterol, we investigated the effect of knockdown of RUNX2 on collagen I expression in PAVICs. An siRNA was designed to specifically knock down porcine RUNX2 in PAVICs. RUNX2 protein expression was indeed decreased by ~50% by RUNX2 siRNA (Fig. 6A,B), indicating effective knockdown of RUNX2. Interestingly, knockdown of RUNX2 prevented the upregulation of collagen I in PAVICs treated with Klotho‐deficient FBS and cholesterol (Fig. 6A,C), suggesting for the first time that RUNX2 is a critical mediator of Klotho deficiency‐induced upregulation of collagen I. In addition, osteocalcin (OCN) protein expression was upregulated in PAVICs treated with Klotho‐deficient serum and cholesterol, which was abolished by knockdown of RUNX2 (Fig. 6A,D). This result suggests that RUNX2 plays a critical role in the upregulation of ECM protein expression due to Klotho deficiency and high cholesterol.

## Discussion

Aortic valve disease, or FAVD, is a leading cause of adult heart disease (Thom *et al*., 2006; Lloyd‐Jones *et al*., 2010) and is the most common form of acquired valvular disease in the USA (Lindroos *et al*., 1993; Baumgartner, 2005; Freeman & Otto, 2005). Unfortunately, due to its unknown etiology, there is currently no cure. The most important finding of this study is that Klotho deficiency promotes formation of AVF in mice fed with a HFD. Our study provides the first experimental evidence that Klotho deficiency is a pathological factor for AVF, which is an important remodeling process that causes aortic valve stiffening, eventually leading to aortic valve calcification and aortic stenosis. It is known that serum levels of Klotho decrease after age 40 (Xiao *et al*., 2004), while the prevalence of aortic stenosis increases with age (Lindroos *et al*., 1993). In this study, we found that the serum level of Klotho was reduced by 50% in *KL* ^*+/−*^ mice (Fig. S2), which mimics the halving of Klotho protein levels in the aged population (Xiao *et al*., 2004), and *KL* ^*+/−*^ mice fed with a HFD may be a natural model of AVF. Klotho homozygous (−/−) mice demonstrate early and extensive aging phenotypes and die before the age of 8 weeks (body weight = 8 g) (Kuro‐o *et al*., 1997). They also develop severe hyperphosphatemia and nonselective soft tissue calcification (Wang & Sun, 2009; Xu & Sun, 2015). For these reasons, Klotho homozygous mice were not used in this study.

It is interesting that Klotho deficiency plus a HFD downregulated valvular AMPKα activity (Fig. 1), although the detailed mechanism remains to be investigated. This is the first study demonstrating that downregulation of AMPKα activity mediates Klotho deficiency‐induced fibrotic formation in aortic valves, which can be abolished by activation of AMPKα by AICAR (Fig. 2). This finding is also significant because it provides a new and important therapeutic strategy for AVF. Recent clinical trials showed that statin failed to attenuate the progression of aortic stenosis (Cowell *et al*., 2005; Houslay *et al*., 2006; Rossebo *et al*., 2008; Chan *et al*., 2010), suggesting that antihyperlipidemia therapy alone is insufficient for treatment of the disease. The findings from the current study suggest that pharmacological activation of AMPKα should be tested for treating FAVD. Aortic valve stenosis, which is the most common valvular disease in the elderly population (Lindroos *et al*., 1993), is associated with a decline in serum levels of Klotho (Xiao *et al*., 2004; Xu & Sun, 2015). Thus, an additional study is warranted for assessing the effect of administration of recombinant Klotho protein on aging‐related aortic stenosis.

Klotho deficiency led to an increase in RUNX2 levels in aortic valves, which was exacerbated by a HFD (Fig. 4). The upregulation of RUNX2 may be mediated by downregulation of AMPKα activity, as it can be abolished by activation of AMPKα by AICAR. The finding that AMPKα regulates RUNX2 is interesting and provides new mechanistic insight into the regulation of RUNX2, a transcription factor that is involved in the osteoblastic transition. RUNX2 regulates the transcription of various genes, including osteocalcin (OCN), *via* binding to the core site of their enhancers or promoters (Viereck *et al*., 2002; Tu *et al*., 2008). Indeed, protein expression of OCN, an ECM protein, was upregulated in *KL* ^*+/−*^ mice, which can be eliminated by silencing of RUNX2 (Fig. 6).

The development of AVD involves phenotypic changes in valvular interstitial cells through the osteogenic pathway (Cheek *et al*., 2012; Leopold, 2012; Nagy *et al*., 2013; Weiss *et al*., 2013). Unexpectedly, no obvious calcification was found in aortic valves in *KL* ^*+/−*^ mice fed with a HFD (Fig. S1). It is anticipated that fibrosis would eventually lead to calcification after a longer period of HFD treatment, because fibrosis may promote aortic valve calcification (Weiss *et al*., 2013).

Klotho directly interacts with valvular interstitial cells and regulates their functions. Indeed, Klotho‐deficient serum upregulated collagen expression in cultured aortic valve interstitial cells, which was abolished by silencing of RUNX2 (Figs 5 and 6). These results demonstrate for the first time that upregulation of RUNX2 is involved in Klotho deficiency‐induced collagen synthesis in aortic valve interstitial cells. Therefore, this study identifies a new pathway that may mediate the stimulatory effect of Klotho deficiency on HFD‐induced AVF as follows: Klotho deficiency 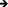 AMPKα 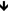 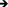 RUNX2 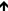 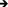 collagen synthesis 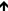 (Fig. S3).

One technical challenge of this study is the limited amount of aortic valve tissue available for molecular assays. We realize the limitation of the IHC assays, which allow only semi‐quantitative analysis. Therefore, we confirmed the IHC result that Klotho deficiency plus cholesterol induces collagen synthesis in cultured porcine aortic valvular interstitial cells (Figs 5 and 6). We further elucidated the molecular pathway in Klotho deficiency‐induced collagen synthesis in cultured valvular interstitial cells (Figs 5 and 6). Although HFD increased plasma levels of cholesterol (data not shown), it may also increase the levels of other lipids. Thus, we realize the limitation of manipulating only cholesterol levels in the cell study, which may partially, but not completely, reproduce the effect of a HFD in animals. We observed that heart function was not altered significantly in *KL* ^*+/−*^ mice fed with HFD for 15 weeks (Fig. S4), which suggests that AVF formation was still at an early stage. The development of aortic stenosis is a slow process, and noticeable changes in heart function would not occur until the late stages of decompensation. We anticipate that longer treatment with a HFD would cause obvious aortic stenosis that would eventually compromise heart function.

### Perspective

This study reveals a previously unidentified role of KL deficiency in promoting the development of HFD‐induced AVF. The promoting effect may be mediated by downregulation of AMPKα activity, which leads to upregulation of RUNX2 and collagen I levels in aortic valves. Therefore, therapeutic activation of AMPKα might be a novel strategy for alleviating arterial stiffening and hypertension.

## Funding

This work was supported by NIH R01 HL118558, DK093403, AG049780, HL122166, HL116863, HL105302, and HL102074. This publication was made possible by NIH Grant Number 9P20GM104934‐06 from the COBRE Program of the National Institute of General Medical Sciences.

## Conflict of interest

None.

## Supporting information


**Fig. S1** Alizarin red staining of aortic valves in *KL* ^*+/−*^ mice fed with a high‐fat diet and treated with 5‐amino‐1‐β‐D‐ribofuranosyl‐imidazole‐4‐carboxamide (AICAR).
**Fig. S2** Western blot analysis of Klotho in the serum.
**Fig. S3** The molecular pathway of the promoting effect of Klotho deficiency on high‐fat‐diet‐induced aortic valve fibrosis.
**Fig. S4** Cardiac output, stroke distance, mean velocity, and mean acceleration of *KL* ^*+/−*^ mice fed with a HFD and treated with AICAR.
**Appendix S1** Methods and Data.
