# Supplementary material for: Deficiency in the anti‐aging gene Klotho promotes aortic valve fibrosis through AMPKα‐mediated activation of RUNX2
Source: Aging Cell. 2016 May 31;15(5):853–60. doi: 10.1111/acel.12494 (PMC5013020; doi:10.1111/acel.12494)
Supplement: Supplementary file 1 — Fig. S1 Alizarin red staining of aortic valves in KL +/− mice fed with a high‐fat diet and treated with 5‐amino‐1‐β‐D‐ribofuranosyl‐imidazole‐4‐carboxamide (AICAR). Fig. S2 Western blot analysis of Klotho in the serum. Fig. S3 The molecular pathway of the promoting effect of Klotho deficiency on high‐fat‐diet‐induced aortic valve fibrosis. Fig. S4 Cardiac output, stroke distance, mean velocity, and mean acceleration of KL +/− mice fed with a HFD and treated with AICAR. Appendix S1 Methods and Data. [file ACEL-15-853-s001.pdf]

Online Supplemental Methods and Data

Deficiency in the Anti-Aging Gene Klotho Promotes Aortic Valve Fibrosis through AMPKα-mediated Activation of RUNX2

Jianglei Chen, Yi Lin, Zhongjie Sun*

Department of Physiology, College of Medicine, University of Oklahoma Health Sciences Center, Oklahoma City, OK73104, USA

Running Title: Klotho and FAVD

Total characters: 36,539; **Total Words**: 6,149

*Address Correspondence to:
Zhongjie Sun, MD, PhD, FAHA
Professor and Vice Chair
Chair, Research Committee
Director, The Robert & Mary Cade Laboratory
BMSB 662A, Box 26901
Department of Physiology, BMSB 662A
College of Medicine
University of Oklahoma Health Sciences Center (OUHSC)
940 Stanton L. Young Blvd.
Oklahoma City, OK 73126-0901
USA
Zhongjie-Sun@ouhsc.edu
Tel. 405-271-2226 x56237
Fax. 405-271-3181

Online Supplemental Methods

Animal studies

Briefly, all mice were housed in cages at room temperature (25±1°C) and provided with laboratory chow (cat. no. 5053, PicoLab) and tap water ad libitum throughout the experiment. This study was carried out according to the guidelines of the National Institutes of Health (NIH) on the Care and Use of Laboratory Animals. Heterozygous Klotho-deficient ($KL^{+/-}$) mice and WT littermates at the age of 6 months were used. Mice were fed with a HFD (Harlan Teklad) containing 21.2% fat (wt/wt) and 1% cholesterol (wt/wt) or a normal diet containing 5% fat and 141 ppm cholesterol. At the end of the 13th week of HFD feeding, AICAR (500 mg/Kg, BIoVision Inc., Milpitas, CA, USA), which is an AMP analog and activator of AMPK (Sriwijitkamol & Musi 2008), was injected (IP) daily for 2 weeks.

Tissue collection

After 2 weeks of AICAR treatment, animals were euthanized with an overdose of ketamine (180 mg/kg body weight) and xylazine (20 mg/kg body weight), and blood was collected with heparin as an anticoagulant. Following blood collection, animals were perfused transcardiacally using heparinized saline. The upper part of the heart along with the ascending aorta was placed in 4% paraformaldehyde (PFA) in phosphate-buffered saline (PBS) for 24 hours and then embedded in paraffin.

Morphological Analysis

A series of 5-µm cross-sections of the aortic valve were cut and stained using the Masson Trichrome Staining Kit (EMS, Hatfield, PA). Immunohistochemical (IHC) procedures were performed as described in our previous studies (Crosswhite *et al.* 2014; Chen *et al.* 2015; Lin & Sun 2015a; Lin & Sun 2015b; Zhou *et al.* 2015). Briefly, IHC staining against AMPKα, phospho-AMPKα (cat. no. SAB4503754, Sigma-Aldrich), RUNX2 (cat. no. ab23981, Abcam), and collagen I (cat. no. ab34710, Abcam) was performed using the rabbit ABC staining system (Santa Cruz Biotechnology). Images of aortic valves were collected at the same exposure conditions under a Nikon Eclipse Ti microscope (10x, 20x, and 40x objective). The fractional

areas of collagen fibrosis components (blue, trichrome staining; brown, IHC staining) in the aortic valve region were obtained using image J software (NIH).

Isolation and Culture of Primary Porcine Aortic Valve Interstitial Cells

Primary porcine aortic valve interstitial cells (PAVICs) were collected from the hearts of female pigs as described (Johnson *et al.* 1987; Butcher & Nerem 2004). Briefly, intact porcine heart with ascending aortas were obtained from a local slaughterhouse (Edmond, OK). The aortic valve was cut from the ascending aorta above the sinuses, and the leaflets were then excised one-third of the distance from the base of the cusp. The leaflets were then serially rinsed in cold PBS with 1% penicillin/streptomycin and 1% amphotericin B. The surfaces of the leaflets were then partially digested with collagenase type II (1000 U/ml; cat. no. 17101015 Life Technologies) for 5 min, after which the endothelium was denuded with gentle scraping. The remaining portions of the leaflets were then minced and digested overnight in fresh collagenase. Undigested tissue pieces were removed with a 70-µM cell strainer, and the cells were seeded into 6-well plates after washing. PAVICs were cultured in DMEM (1 g/L glucose, Invitrogen), supplemented with 10% fetal bovine serum (FBS), 1% penicillin/streptomycin, and 1% L-glutamine. Cultures were fed every 48 h and split 1:3 at confluence. Cultures were used for experiments between passages 4 and 8. Confluent cells were incubated with Dulbecco's modified Eagle's medium (DMEM) containing 1 mM glucose and 2% FBS or 2% KL-deficient FBS in the presence of water-soluble cholesterol (cat. no. C4951, 10µM, Sigma-Aldrich) and AICAR (100µM) for 24–48 hours. KL-deficient FBS was generated using a Pierce Direct IP kit (cat. no. 26148, Pierce) with an antibody against Klotho (Cat # AF1819, R&D Systems) as we described recently (Fan & Sun 2016). The Klotho protein level was reduced by 50% in FBS (Fan & Sun 2016). Cells were collected in RIPA buffer containing a protease inhibitor cocktail and a phosphatase inhibitor cocktail (Research Products International Corp., Mount Prospect, IL USA).

Immunofluorescence Analysis

Cells were fixed with 4% PFA for 10 minutes and then permeabilized with 0.2% Triton X-100 in PBS for 10 minutes. After blocking with 1% BSA, 0.3 M glycine in PBST for 1 hour, cells were incubated with a specific primary antibody at 4°C overnight. After washing with PBST, the cells were then incubated with Alexa Fluor secondary antibodies (cat. no. A-11008, Invitrogen,

Carlsbad, CA) at dilutions of 1:500 for 1 hour at room temperature in the dark. The slides were mounted with UltraCruz mounting medium containing 4',6-diamidino-2-phenylindole (cat. no. sc24941, DAPI, Santa Cruz Biotechnology, TX). Fluorescent images were taken with an IX73 microscope (Olympus, Tokyo, Japan).

Western blotting

Western blotting was performed as described in our previous studies (Goetz *et al.* 2010; Belting *et al.* ; Lin *et al.* 2016). Briefly, cells were lysed in RIPA buffer containing protease inhibitor cocktail, 2.5 mM sodium pyrophosphate, 1 mM β-glycerolphosphate, 2 mM sodium vanadate, 1 mM EDTA, and 1 mM EGTA. The protein concentration was measured using the Pierce BCA assay (Thermo Scientific). The lysates (30 µg protein/well) under reduced conditions were directly subjected to SDS-PAGE (4–15% Tris-HCL precast gel, Bio-Rad) followed by western blotting with antibodies against collagen I (cat. no. ab34710, Abcam), RUNX2 (cat. no. ab23981, Abcam), pAMPKα (Thr172; cat. no.2531, Cell Signaling), AMPKα (cat. no. 2193, Cell Signaling), and osteocalcin (cat. no. AB10911, Millipore). The blot was then rinsed and reprobed with antibodies against β-actin (cat. no. ab8227, Abcam) for the loading control.

Reference

(2011). Drugs used in child maltreatment. *Prescrire international*. **20**, 236-237.

Belting M, Almgren P, Manjer J, Hedblad B, Struck J, Wang TJ, Bergmann A, Melander O (2012). Vasoactive peptides with angiogenesis-regulating activity predict cancer risk in males. *Cancer epidemiology, biomarkers & prevention : a publication of the American Association for Cancer Research, cosponsored by the American Society of Preventive Oncology*. **21**, 513-522.

Butcher JT, Nerem RM (2004). Porcine aortic valve interstitial cells in three-dimensional culture: comparison of phenotype with aortic smooth muscle cells. *The Journal of heart valve disease*. **13**, 478-485; discussion 485-476.

Chen K, Zhou X, Sun Z (2015). Haplodeficiency of Klotho Gene Causes Arterial Stiffening via Upregulation of Scleraxis Expression and Induction of Autophagy. *Hypertension*. **66**, 1006-1013.

Crosswhite P, Chen K, Sun Z (2014). AAV delivery of tumor necrosis factor-alpha short hairpin RNA attenuates cold-induced pulmonary hypertension and pulmonary arterial remodeling. *Hypertension*. **64**, 1141-1150.

Fan J, Sun Z (2016). The Anti-aging Gene Klotho Regulates Proliferation and Differentiation of Adipose-derived Stem Cells. *Stem cells*.

Goetz R, Nakada Y, Hu MC, Kurosu H, Wang L, Nakatani T, Shi M, Eliseenkova AV, Razzaque MS, Moe OW, Kuro-o M, Mohammadi M (2010). Isolated C-terminal tail of FGF23 alleviates

hypophosphatemia by inhibiting FGF23-FGFR-Klotho complex formation. *Proceedings of the National Academy of Sciences of the United States of America*. **107**, 407-412.
Johnson CM, Hanson MN, Helgeson SC (1987). Porcine cardiac valvular subendothelial cells in culture: cell isolation and growth characteristics. *Journal of molecular and cellular cardiology*. **19**, 1185-1193.
Lin Y, Chen J, Sun Z (2016). Antiaging Gene Klotho Deficiency Promoted High-Fat Diet-Induced Arterial Stiffening via Inactivation of AMP-Activated Protein Kinase. *Hypertension*. **67**, 564-573.
Lin Y, Sun Z (2015a). Anti-aging Gene Klotho Attenuates Pancreatic beta Cell Apoptosis in Type I Diabetes. *Diabetes*.
Lin Y, Sun Z (2015b). In Vivo Pancreatic beta-Cell-Specific Expression of Antiaging Gene Klotho: A Novel Approach for Preserving beta-Cells in Type 2 Diabetes. *Diabetes*. **64**, 1444-1458.
Reddy AK, Jones AD, Martono C, Caro WA, Madala S, Hartley CJ (2005). Pulsed Doppler signal processing for use in mice: design and evaluation. *IEEE transactions on bio-medical engineering*. **52**, 1764-1770.
Sriwijitkamol A, Musi N (2008). Advances in the development of AMPK-activating compounds. *Expert opinion on drug discovery*. **3**, 1167-1176.
Zhou X, Chen K, Wang Y, Schuman M, Lei H, Sun Z (2015). Antiaging Gene Klotho Regulates Adrenal CYP11B2 Expression and Aldosterone Synthesis. *J. Am. Soc. Nephrol.*

Online Supplemental Data

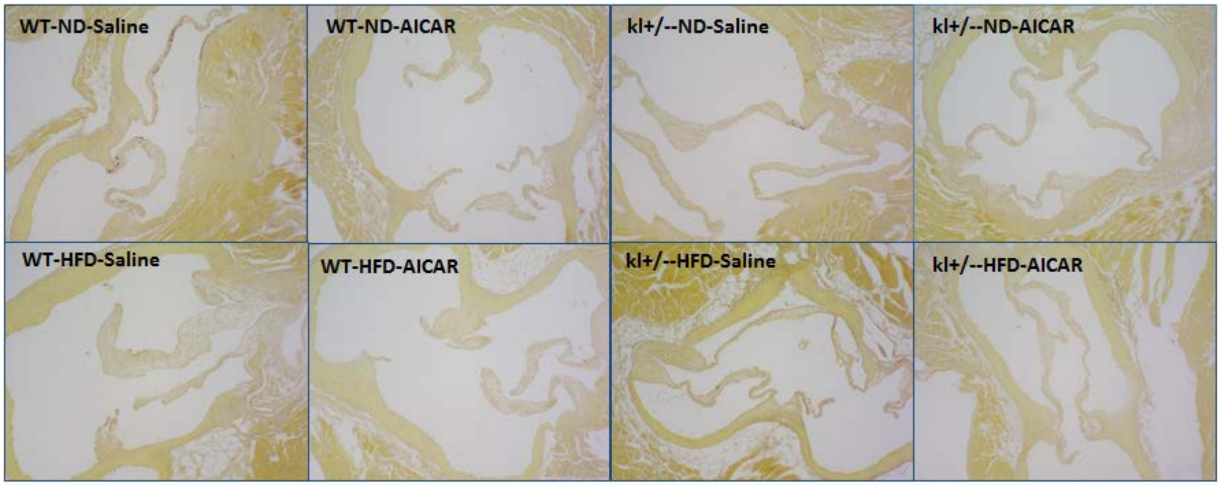

Supplemental Figure 1. Alizarin red staining of aortic valves in $KL^{+/-}$ mice fed with a high-fat diet and treated with 5-amino-1-β-D-ribofuranosyl-imidazole-4-carboxamide (AICAR).

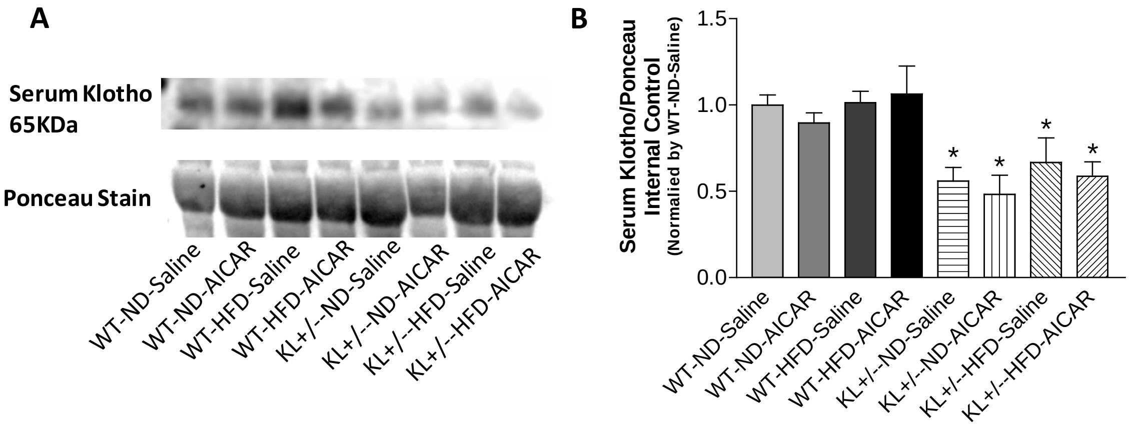

Supplemental Figure 2. Western blot analysis of Klotho in the serum. Ponceau staining was used as a loading control. Serum levels of Klotho were decreased by 50% in KL+/- mice *vs.* WT mice, confirming haplodeficiency of Klotho. HFD or AICAR treatments did not affect serum levels of Klotho significantly. N=3. Data = means±SEM. Data were calculated as fold change of the WT-ND-Saline group. *$p<0.05$ *vs.* WT-ND-Saline.

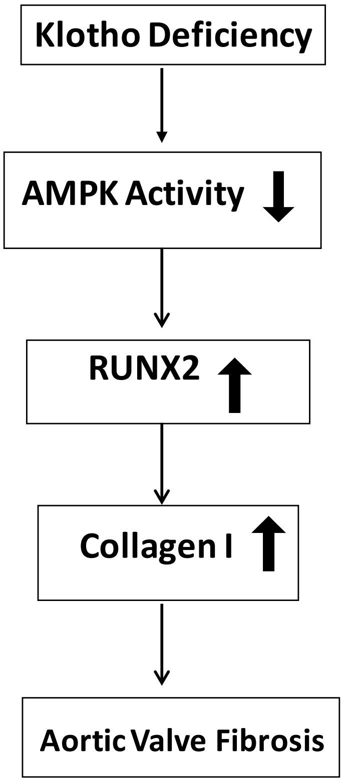

Supplemental Figure 3. The molecular pathway of the promoting effect of Klotho deficiency on high-fat-diet-induced aortic valve fibrosis. RUNX2, runt-related transcription factor 2. AMPKα, AMP-activated protein kinase.

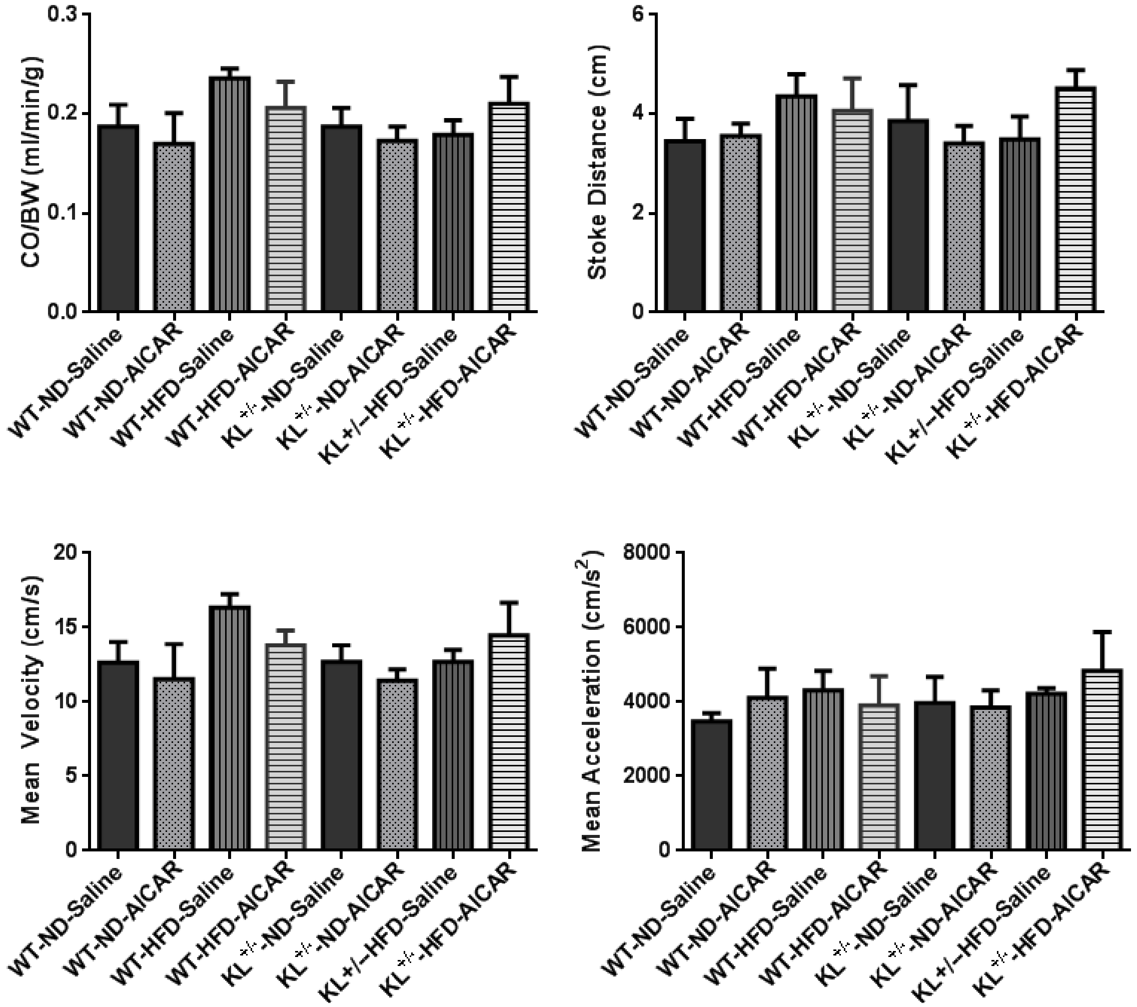

Supplemental Figure 4. Cardiac output, stroke distance, mean velocity, and mean acceleration of *KL*$^{+/-}$ mice fed with a HFD and treated with AICAR.

Heart Function Data Collected Using Pulsed Doppler

Aortic flow was measured using a Doppler Signal Processing Workstation (DSPW, Indus Instruments, Houston, TX, USA) (Reddy *et al.* 2005). Briefly, all mice were anesthetized with ketamine (90 mg/kg body weight) and xylazine (10 mg/kg body weight) *via* IP injection. Each mouse was taped supine to electrocardiogram (ECG) electrodes on a heated procedure board with a constant temperature of 37 °C (Indus Instruments). A 2-mm diameter, 10-MHz Doppler

probe was used to measure aortic flow. Heart function data was calculated using the manufacturer’s DSPW software.
